# Circadian lighting effect for inpatients with schizophrenia: A prospective cohort study

**DOI:** 10.1002/brb3.70003

**Published:** 2024-08-25

**Authors:** Ya‐Chi Tsai, Jwo‐Huei Jou, Ching‐Chi Hsu, Ming‐Chang Shih, Luke The, Dipanshu Sharma

**Affiliations:** ^1^ Department of International Intercollegiate PhD Program National Tsing Hua University Hsinchu Taiwan, ROC; ^2^ Board of Directors, Wizcare Medical Corporation Aggregate Taichung Taiwan, ROC; ^3^ Department of Materials Science and Engineering National Tsing Hua University Hsinchu Taiwan, ROC

**Keywords:** cognitive function, lighting chronotherapy, psychopathology, schizophrenia

## Abstract

**Objectives:**

In schizophrenia, nonspecific lighting likely causes sleep timing disturbances, leading to distress and poorer clinical status. However, the effect of exposure to circadian lighting on psychopathology outcome in schizophrenia remains unknown. Hence, this study aimed to develop such an intervention and investigate its impact on schizophrenia.

**Methods:**

Twenty schizophrenia patients at a psychiatric nursing institute were monitored over 10 weeks, with assessments using the Brief Psychiatric Rating Scale (BPRS) and Mini‐Mental State Examination (MMSE) conducted at baseline, weeks 3 (T1), 7 (T2), and 10 (T3).

**Results:**

Circadian lighting significantly improved BPRS scores between T1–T2 (*p* < .05) and T1–T3 (*p* < .001), with affectivity scores also showing significant enhancements postintervention. Notably, female participants exhibited substantial improvements in BPRS scores from T1 to T3 (*p* < .01), while male participants demonstrated significant gains in MMSE scores from T1 to T2 (*p* < .01).

**Conclusions:**

Circadian lighting presents a promising intervention for improving psychiatric outcomes in schizophrenia, with distinct benefits observed across different psychopathological aspects and genders. These findings underscore the potential of lighting chronotherapy in psychiatric clinical practice and warrant further exploration in related research.

## Graphical abstract

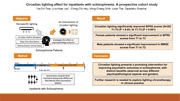

## 1 INTRODUCTION

Sleep and circadian rhythm disorders (SCRD) are prevalent in up to 80% of individuals with schizophrenia (Krysta et al., 2017). Research links these disruptions with exacerbated psychopathology (Afonso et al., 2011), cognitive impairments (Bromundt et al., 2011), and reduced quality of life (Reeve et al., 2019). Additionally, chronic sleep disturbances and circadian misalignment are associated with broader health issues such as cardiometabolic diseases and social isolation in both the schizophrenic and general populations (Scott et al., 2021).

Recent studies underscore the importance of chronotype, showing its significant impact on schizophrenia's severity and management, which current clinical practices often neglect. Notably, Kirlioglu and Balcioglu (2020) and Linke and Jankowski (2021) have identified chronotype variations as a key factor in the condition's clinical outcomes, while Balcioglu et al. (2022) highlight the wide‐reaching implications of these circadian disturbances across various health conditions.

Light‐based interventions, or “chronotherapies,” have demonstrated effectiveness across psychiatric disorders, improving cognitive function and sleep quality through structured exposure to bright light during the day and darkness at night (Gottlieb et al., 2019; Parry & Maurer, 2003). Despite their potential, the application of these therapies in clinical settings, particularly for schizophrenia, is limited. This is partly due to the stagnant design of psychiatric units over the past five decades, which has hindered the advancement of treatment methods and environmental factors (Kallestad et al., 2012).

This study aims to investigate the benefits of a dynamic lighting system designed to mimic natural light patterns, thereby regulating circadian rhythms in schizophrenia patients. Simulation studies, such as those by Skeldon et al. (2022) and Van den Berg (2005), suggest that consistent light exposure at suitable intensities can stabilize circadian rhythms, providing a practical light‐based intervention.

However, operationalizing such lighting designs in clinical and residential care settings remains in its early stages, with few established practices for their implementation (Chromaviso Research in Circadian Lighting & User Evidence, 2023). Prior pilot projects have been limited in scope and have only partially addressed these challenges (Nagare et al., 2019; Vethe et al., 2021).

Given these gaps, our research focuses on developing and evaluating an effective circadian rhythm simulation lighting (CRSL) system. This system includes bright light exposure during the day, dim blue‐depleted light after dusk, and darkness at night. We hypothesize that this tailored CRSL system will significantly improve circadian rhythm synchronization, enhance cognitive function, and reduce psychopathology symptoms in inpatients with schizophrenia.

By addressing the need for scalable, economically feasible, and effective interventions, this study aims to advance our understanding of nonpharmacological chronotherapies and their potential impact on schizophrenia management.

## 2 MATERIALS AND METHODS

### 2.1 Study design

This research examined the effects of a Circadian Rhythm‐Simulating Lighting (CRSL) system installed in patient bedrooms at the Wizcare Psychiatric Nursing Institute in Taiwan, highlighting a significant technological advancement by incorporating adjustable color temperature and brightness in indoor lighting from morning to evening (6:30–21:00 h). This prospective, interventional, and observational study was devised to investigate changes in the psychopathological states and cognitive functions of individuals diagnosed with schizophrenia, pre and post the implementation of the lighting intervention.

### 2.2 Participant selection

**FIGURE 1:**
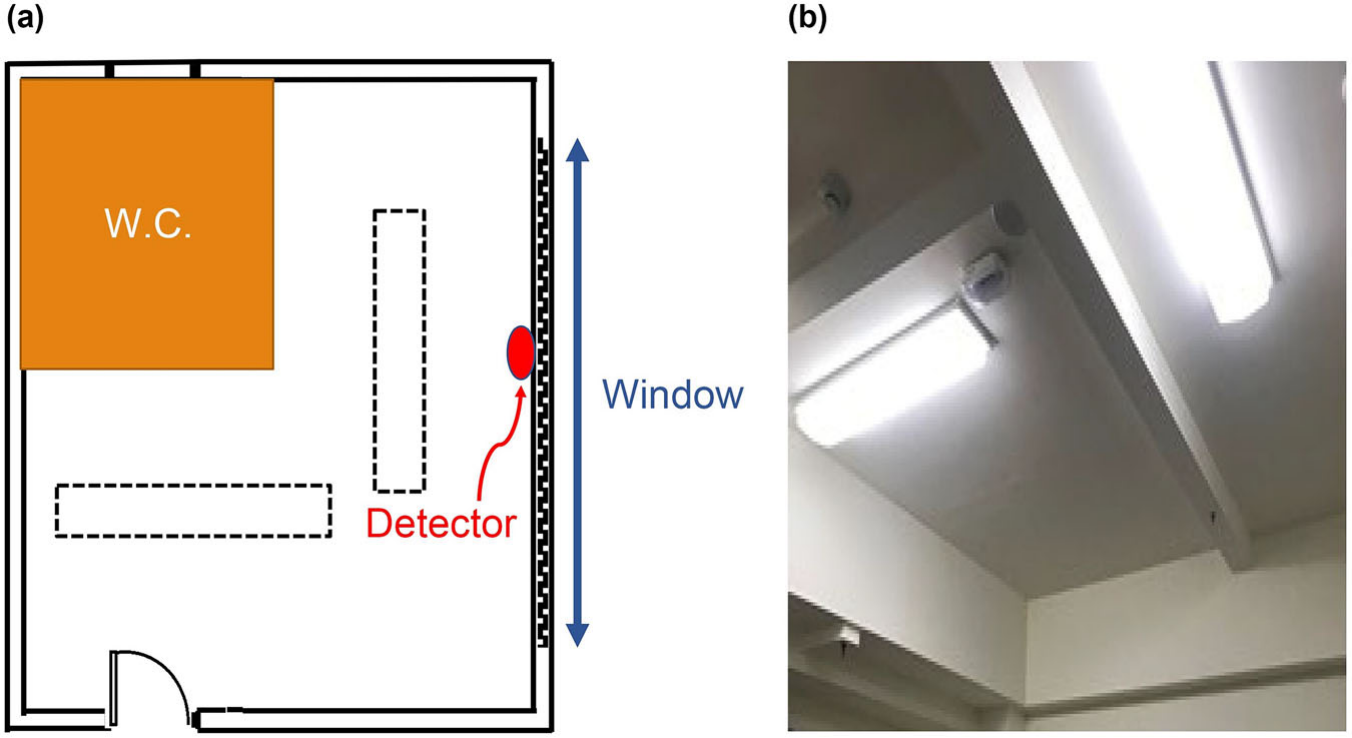
(a) Schematic illustration and (b) original standard lighting with a 5400K fluorescent lamp and a 200 L× reaching detector illuminance.

**FIGURE 2:**
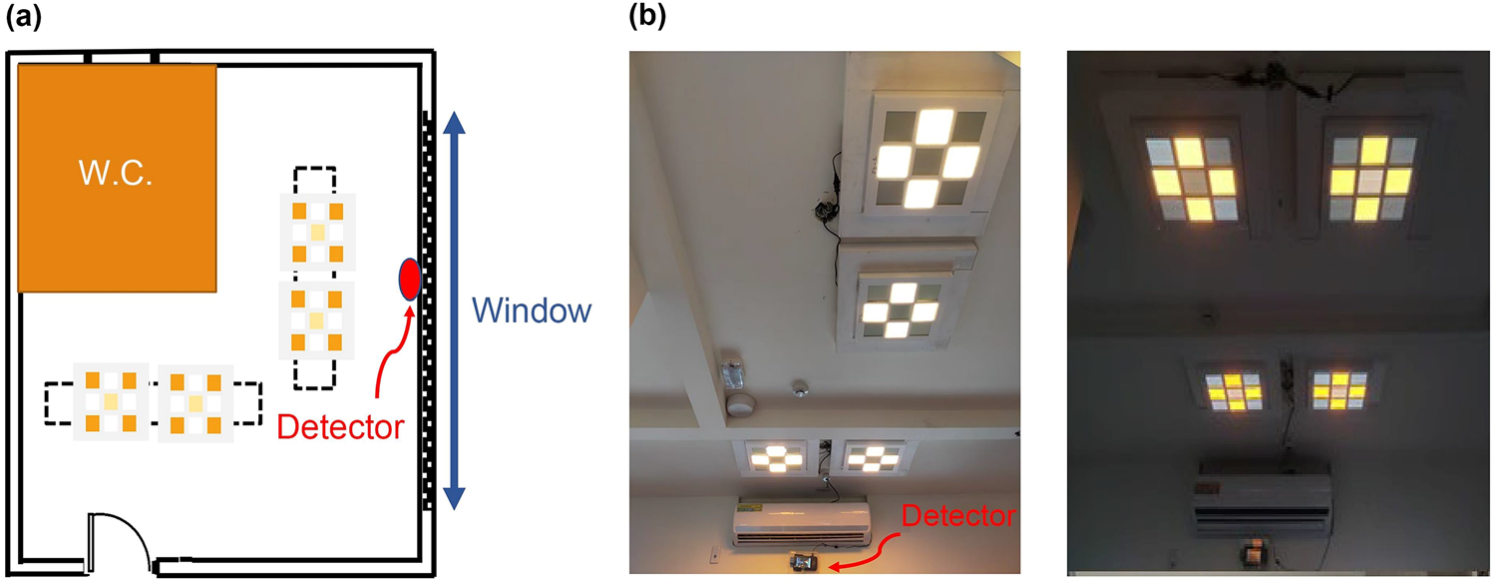
(a) Schematic illustration and (b) photo of the illumination setting in the room of patients with a lumen detector installed underneath the air conditioner. Circadian rhythm simulation lighting with a color temperature of 4000K (130 L×, left) and 2000K (14 L×, right).

The investigation was restricted to inpatients diagnosed with schizophrenia within a psychiatric unit, ensuring a controlled environment where daily routines such as sleep, wake, and meal times were standardized. The study encompassed patients residing in one of six rooms, with a capacity of four individuals per room, segregating female patients to the fourth floor and male patients to the third for privacy and comfort. Participants were initially exposed to standard ambient lighting conditions featuring a 5400 K fluorescent lamp and an illuminance level of 200 lx (Figure 1), for a minimum duration of six months before transitioning to the CRSL environment (Figure 2) in the third week of the study. This selection criterion aimed to minimize circadian rhythm disruptions by maintaining consistent environmental time cues.

Inclusion criteria were meticulously defined to include individuals formally diagnosed with schizophrenia according to DSM‐IV criteria, aged 40 and above, with a history of the condition spanning at least 2 years. Participants were required to be deemed stable by their consulting psychiatrist, hospitalized for a duration exceeding three months, undergoing inpatient treatment, without any changes to their primary antipsychotic medication or sleep aids in the preceding month, and capable of understanding and consenting to participate in the study.

Exclusion criteria encompassed patients with significant neuropsychiatric or physical health issues or those considered unsuitable for the study by their psychiatrists. Out of 21 initial participants, one withdrew due to neuropsychiatric complications, leaving 20 subjects for final analysis. This patient developed symptoms indicative of a manic episode, which we attributed to an underlying bipolar disorder, becoming evident in the fifth week of the intervention (March 19).

### 2.3 Data collection

**FIGURE 3:**
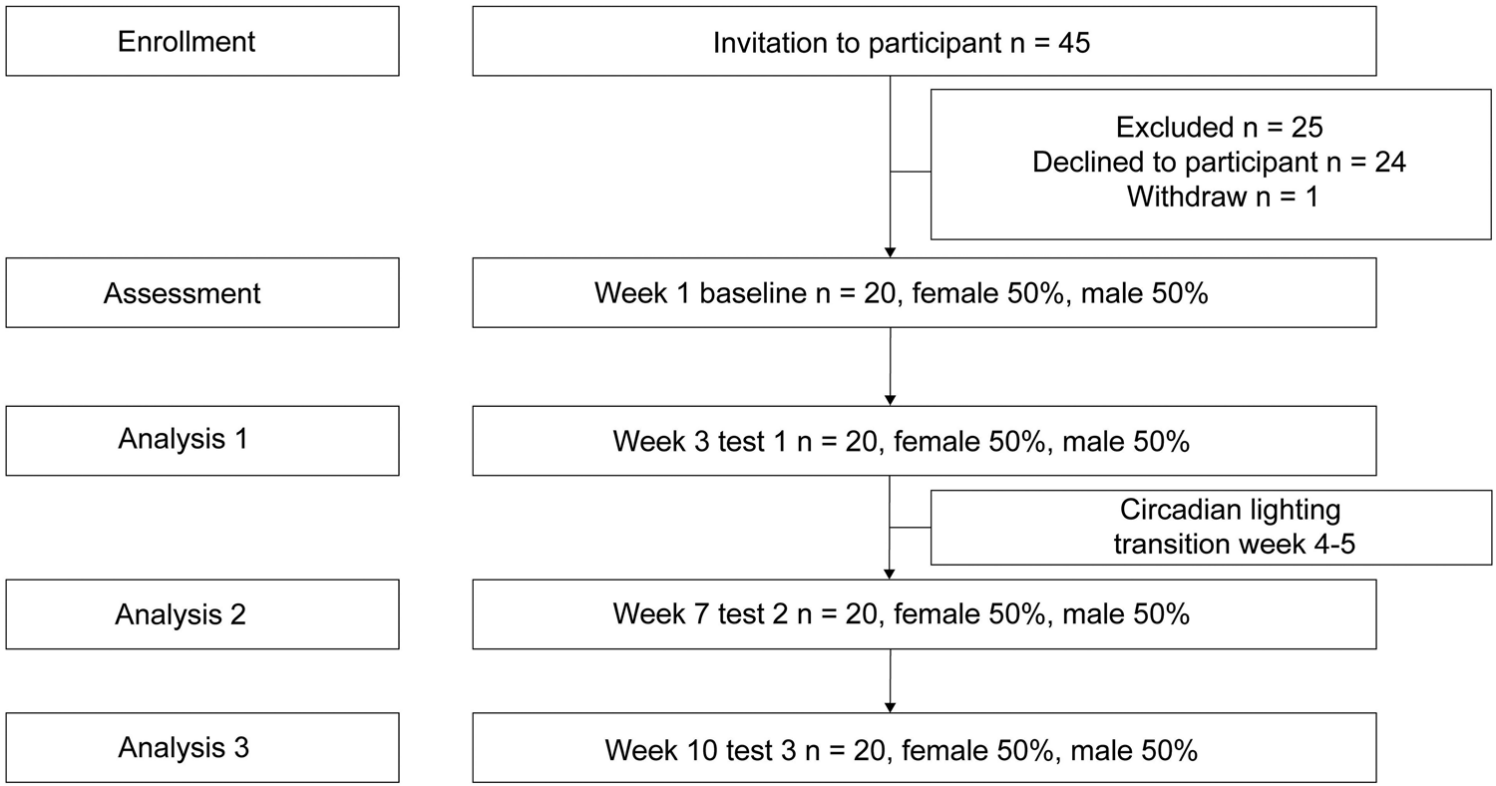
Flowchart of the study design showing the timeline of baseline evaluation, intervention assessments in the 3rd, 7th, and 10th weeks, and the washout transition period for treatment scheduled during the 4th and 5th weeks. The study included 20 patients.

Data collection was structured around four key timelines (Figure 3): baseline (BL, week 1), test phase one (T1, week 3), a washout transition period (weeks 4 and 5), test phase two (T2, week 7, post 2 weeks of treatment), and test phase three (T3, week 10, post 5 weeks of treatment). Qualified psychiatrists conducted assessments using the Brief Psychiatric Rating Scale (BPRS) for psychiatric disorders and the Mini‐Mental State Examination (MMSE) for cognitive functions during the inpatients' routine hospital visits. Demographic and clinical data, including gender, age, educational background, marital status, duration of hospitalization, and medication details, were provided by the primary caregivers

### 2.4 Ethics and informed consent

The ethical framework of the study was rigorously outlined, with all participants receiving comprehensive written information about the research prior to enrollment, ensuring informed consent and adherence to the Declaration of Helsinki principles. The Institutional Review Board of Mackay Memorial Hospital in Taiwan (reference no.: 19MMHIS298e) granted ethical approval. Participants and their families were assured of their anonymity and the right to withdraw from the study at any point without repercussions. To maintain confidentiality, participants were identified through unique codes rather than names, and no ethical concerns were encountered throughout the research duration.

Recruitment was conducted by psychiatric nurses trained in the research protocol to ensure uniformity in data collection, with the principal investigator ensuring protocol adherence and the evaluator demonstrating high interrater reliability. The intervention was delivered in accordance with established guidelines by the first author, who was adequately trained in the procedures.

### 2.5 Measurement techniques and instruments

#### 2.5.1 Assessment of psychopathology: Brief Psychiatric Rating Scale (BPRS)

The BPRS, a widely acknowledged tool for assessing psychopathology in schizophrenia patients (Andreasen et al., 2005), comprises 18 symptom constructs, with assessments typically requiring 20−30 min to complete through interviews and subsequent scoring. It employs a Likert scale ranging from 1 (not present) to 7 (extremely severe), with an alternative 0−6 scale in newer versions (Onu & Ohaeri, 2023); this study utilized the 1−7 scale. The BPRS categorizes symptoms into subscales, including Positive Symptoms (such as unusual thought content and hallucinatory behavior), Negative Symptoms (like blunted affect and emotional withdrawal), Affectivity (covering aspects like anxiety and depressive mood), Resistance (including hostility and uncooperativeness), and Activation (which encompasses excitement and tension), as outlined by Shafer (2005). The attending psychiatrists utilized the BPRS and its subscales for evaluating the severity of psychiatric symptoms in participants.

#### 2.5.2 MMSE

Cognitive impairments are increasingly recognized as central to schizophrenia, with the MMSE serving as a predominant tool for cognitive assessment in this context, praised for its validated use and well‐established psychometric properties (Bowie & Harvey, 2005; Folstein et al., 1975). This 30‐point test evaluates cognitive status across various domains, including orientation, attention, recall, language, and visuospatial abilities, albeit with limitations in assessing executive functions. Despite these limitations, its extensive application in both clinical and research settings underscores its utility (De Leon et al., 1998).

#### 2.5.3 CRSL instruments

Given the growing body of evidence linking light exposure to mood and well‐being, this study leveraged light as a therapeutic intervention (Beauchemin & Hays, 1996; Scott et al., 2019), especially considering the crucial role of short‐wavelength and blue light in sustaining alertness and cognitive performance throughout the day (Wahl et al., 2019). However, exposure to blue light, particularly before sleep, can disrupt sleep quality and circadian rhythms (Castro et al., 2011). The study introduced a sunlight‐style circadian lighting instrument (Chen et al., 2007; Jou et al., 2012; Langsrud et al., 2016), capable of independent adjustments in color temperature and illuminance, designed to support circadian health by increasing bright, white light exposure in the morning and reducing light intensity and blue light exposure toward bedtime.

**FIGURE 4:**
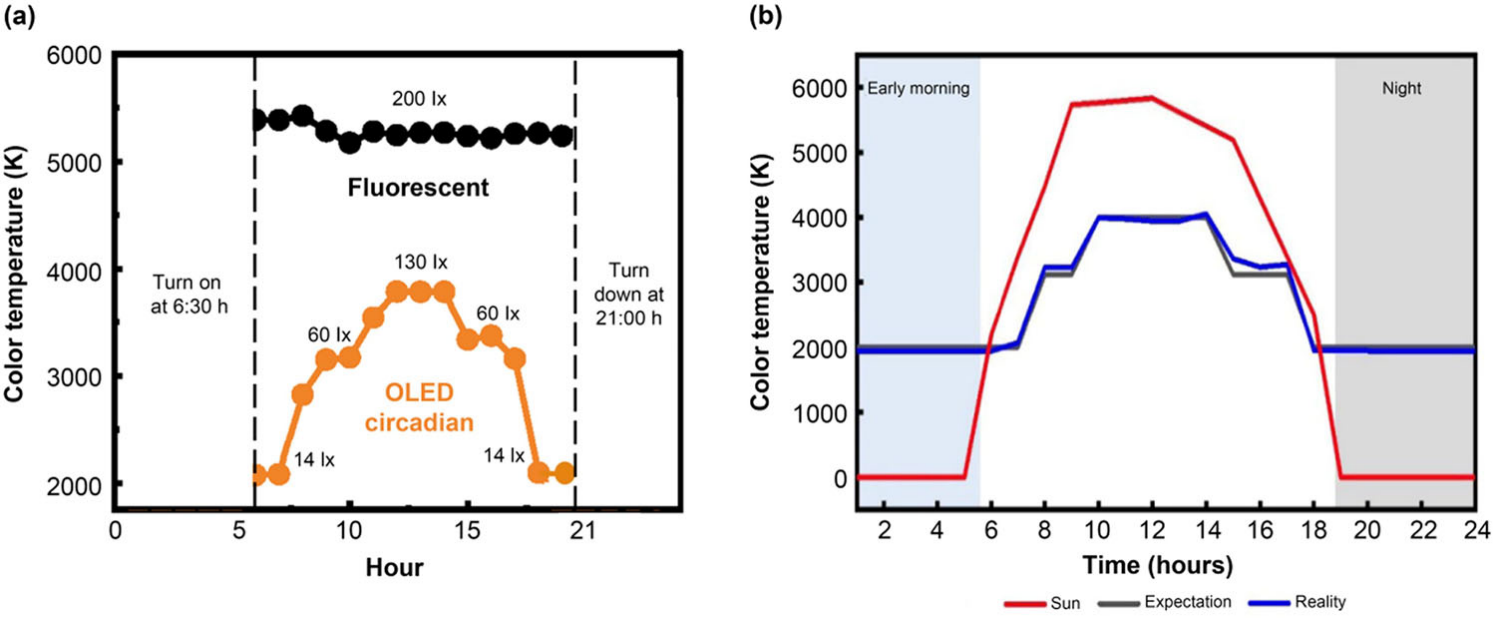
(a)Time‐dependent illuminance and color temperature of the circadian lighting in contrast to the standard lighting (brightness, 200 photopic lux; color temperature, 5400 K). (b) Time‐dependent sunlight and simulated design work, and simulation of circadian rhythm lighting expectation versus the real measured data.

The ambient lighting intervention orchestrated a dynamic lighting environment, with early morning settings at 2000 K/14 lx transitioning to 4000 K/130 lx around noon, and reverting to 2000 K/14 lx in the evening, ultimately leading to a complete blackout overnight to simulate natural darkness (Figure 4). The bedrooms, identical in size and layout, featured east‐facing windows to maximize morning sunlight exposure. Lighting conditions were quantified using a portable spectrometer (SRI‐100, Figure 2; IBOSON Technology Co., Ltd.), which measured illuminance, color temperature, and spectral distribution, ensuring an accurate representation of the light environment experienced by the patients.

Furthermore, the study accounted for daily nonpharmacological interventions, such as ergo therapy, physiotherapy, and psychotherapy, by consulting patients' medical records, enriching the dataset with comprehensive treatment information and allowing for a nuanced understanding of the intervention's impact

#### 2.5.4 Statistical analyses

Statistical analysis involved descriptive statistics (*n*, %, mean, standard error) at baseline, with participant characteristics evaluated via paired *t*‐tests and Kruskal–Wallis tests (Table 1). Generalized estimating equations were employed to assess intervention effects amidst repeated measures (Zeger & Liang, 1986), while repeated‐measures ANOVA facilitated the comparison of MMSE and BPRS scores over time. Statistical significance was determined by a two‐tailed *p*‐value < .05, utilizing SPSS (version 29.0) for all analyses.

**TABLE 1:** Demographic and clinical characteristics of all participants.

Characteristics	BPRS scores MDN		*P*_1_	
Sex			0.38	
Male (*n* = 10, 50%)	32			
Female (*n* = 10, 50%)	28			
Education			***P*_2_**	
1. Completed primary school (*n* = 4, 20%)	27.5		0.3	
2. Completed upper secondary school (*n* = 12, 60%)	28			
3. Completed higher education (*n* = 3, 15%)	34			
Marital status			0.4	
1. Unmarried (*n* = 14, 70%)	28			
2. Married/cohabitant (*n* = 1, 5%)	25			
3. Divorced (*n* = 5, 25%)	35			
	**Mean (SD)**	**MDN**	**MIN**	**MAX**
Age	49.5 (6.3)	48.5	40	66
Duration since 1st visit	17.75 (10.7)	18.5	2	36
MMSE scores	27.3 (2.7)	27.5	22	30
BPRS scores (total)	29.5 (6.4)	28.5	19	44
BPRS scores (affectivity)	8.1 (4.8)	7.5	4	21
BPRS scores (positive symptoms)	4.9 (1.4)	4	4	9
BPRS scores (negative symptoms)	8 (3.3)	8	3	14
BPRS scores (resistance)	3.85 (1.5)	3	3	9
BPRS scores (activation)	3.45 (0.9)	3	3	6

MDN, median; MIN, minimum; MAX, maximum; BPRS, Brief Psychiatric Rating Scale; MMSE, Mini‐Mental State Examination; *P*_1_, Mann–Whitney *U* test; *P*_2_, Kruskal–Wallis test.

## 3 RESULTS

### 3.1 Participant demographics and clinical profiles

An analysis of the initial demographic and clinical data of the participants revealed a uniform distribution across the cohort, as outlined in Table 1, laying the groundwork for subsequent evaluations.

### 3.2 Sex‐based clinical characteristics

Baseline clinical assessments, including MMSE and BPRS scores, were detailed in Table 2, comparing male and female participants diagnosed with schizophrenia. The cohort comprised an equal number of female participants (50%), with significant sex‐based differences noted only in the affectivity subscale of the BPRS, where females exhibited higher mean scores than males, suggesting more pronounced affective symptoms in females at the study's outset.

**TABLE 2:** Descriptive statistics of baseline BPRS scores in female and male participants.

Characteristics	Sex	Mean	SD	MDN	MIN	MAX	Skewness	Kurtosis	*p* Value	Cohen's *d*
MMSE scores	F	28	2.6	29	23	30	−0.71	−0.78	>.05	.37
	M	27	2.8	27	22	30				
BPRS scores (total)	F	31.1	7.2	32	21	44	0.27	0.27	>.05	.46
	M	27.9	6.7	28	19	35				
BPRS scores (affectivity)	F	10	5.5	8	4	21	1.39	1.57	<.05*	.84
	M	6.2	3.2	4	4	12				
BPRS scores (positive symptoms)	F	5	1.7	4	4	9	1.7	2.52	>.05	.14
	M	4.8	1.1	4	4	7				
BPRS scores (negative symptoms)	F	7.5	3.3	7.5	3	13	0.02	−0.94	>.05	.3
	M	8.5	3.4	9	3	14				
BPRS scores (resistance)	F	4.3	1.8	4	3	9	2.54	7.83	>.05	.65
	M	3.4	0.8	3	3	5				
BPRS scores (activation)	F	3.3	0.95	3	3	6	2.24	4.22	>.05	.31
	M	3.6	0.97	3	3	6				

*Note*: BPRS, Brief Psychiatric Rating Scale (in total: 18 items; score range: 7−126), and subscales including affectivity (score range: 4−28), positive symptoms (score range: 4−28); negative symptoms (score range: 3−21), resistance (score range: 3−21), and activation (score range: 3−21). *p* Values obtained by Mann–Whitney *U* test.

SD, standard deviation; MDN, median; MIN, minimum; MAX, maximum; Skewness and Kurtosis, a distribution of data set.

The * occuring in Table 2 provides the statistical significance which supported the content, explainging that females exhibited higher mean scores than males in the affectivity subscales of the BPRS.

### 3.3 Investigative findings

#### 3.3.1 CRSL intervention outcomes

**FIGURE 5:**
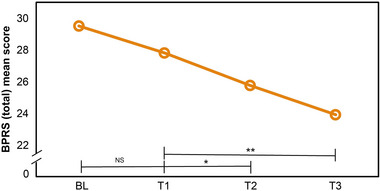
BPRS (total) mean scores of all 20 participants at BL, T1, T2, and T3. After transition to circadian lighting treatment, the BPRS (total) mean scores improved significantly at T1–T2 and T2–T3. BPRS, Brief Psychiatric Rating Scale; BL, baseline (week 1); T1, test 1 (week 3); T2, test 2 (week 7); T3, test 3 (week 10); NS, not significant. *p < .05, **p < .001.

The impact of the Circadian Rhythm‐Simulating Lighting (CRSL) intervention on psychopathological measures was assessed using the BPRS. A noteworthy improvement in BPRS total scores was observed from the initial to the subsequent assessments (*p* < .05, T1 to T2 and *p* < .001, T1 to T3), with significant enhancements indicating the intervention's efficacy (Figure 5). The BPRS subscales highlighted specific improvements in affectivity between the baseline and follow‐up periods under the CRSL intervention (*p* < .05) at T1–T2 and T1–T3, while no significant changes were detected in other subscales postintervention (Table 3).

**TABLE 3:** MMSE, BPRS (total), and BPRS (subscale) mean scores of all participants (*n* = 20).

	BPRS scores of all participants (*n* = 20)						
Characteristics	BL Mean (SD)	T1 Mean (SD)	T2 Mean (SD)	T3 Mean (SD)	BL–T1 *p*	T1–T2 *p*	T1–T3 *p*
MMSE scores	27.3 (2.7)	28.2 (2.1)	28.7 (2.3)	29.0 (1.6)	.16	.37	.09
BPRS scores (total)	29.5 (6.4)	27.8 (5.6)	25.6 (5.0)	23.9 (5.8)	.17	.02*	.001**
BPRS scores (affectivity)	8.1 (4.8)	8.1 (4.4)	7.1 (3.8)	6.1 (3.8)	1	.049*	.03*
BPRS scores (positive symptoms)	4.9 (1.4)	4.9 (1.9)	4.7 (1.3)	4.4 (1.0)	1	.48	.33
BPRS scores (negative symptoms)	8.0 (3.3)	7.0 (3.3)	6.7 (3.2)	5.7 (2.8)	.049*	.56	.06
BPRS scores (resistance)	3.9 (1.5)	3.45 (0.7)	3.4 (0.7)	3.6 (0.9)	.31	.54	.33
BPRS scores (activation)	3.5 (0.9)	3.2 (0.6)	3.1 (0.2)	3.1 (0.2)	.17	.19	.33

*Note*: All data are presented as mean (SD, standard deviation), where *P*_1_ was obtained by one‐way repeated measures ANOVA; *p < .05; **p < .001.

BPRS, Brief Psychiatric Rating Scale; MMSE, Mini‐Mental State Examination; BL, baseline (week 1); T1, test 1 (week 3); T2, test 2 (week 7); T3, test 3 (week 10).

**FIGURE 6:**
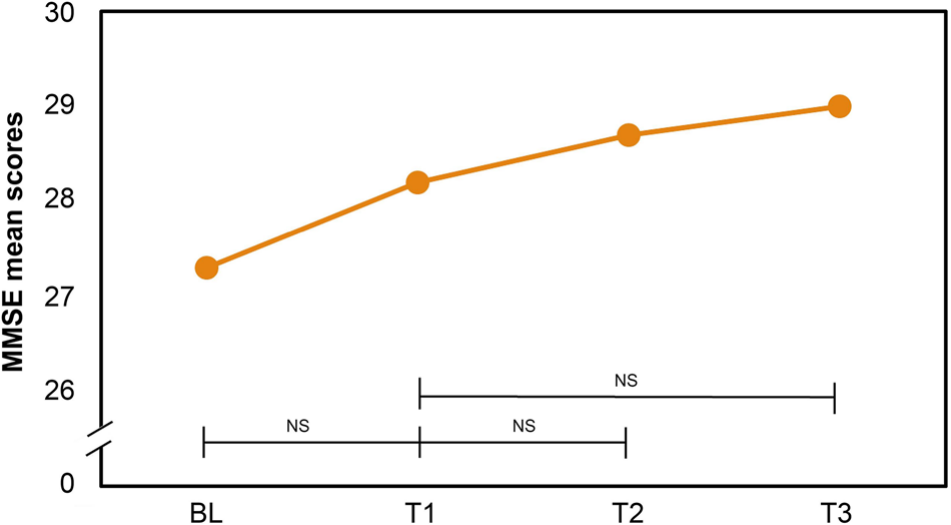
MMSE mean scores of all 20 participants at BL, T1, T2, and T3. After transition to circadian lighting treatment, no significant difference was observed at BL–T1, T1–T2, and T1–T3. MMSE, Mini‐Mental State Examination; BL, baseline (week 1); T1, test 1 (week 3); T2, test 2 (week 7); T3, test 3 (week 10); NS, not significant.

Cognitive function, gauged through the MMSE, did not show significant changes across the study timeline for the entire participant pool, as depicted in Table 3 and Figure 6.

#### 3.3.2 Sex‐differential responses to CRSL

##### Psychopathology

**FIGURE 7:**
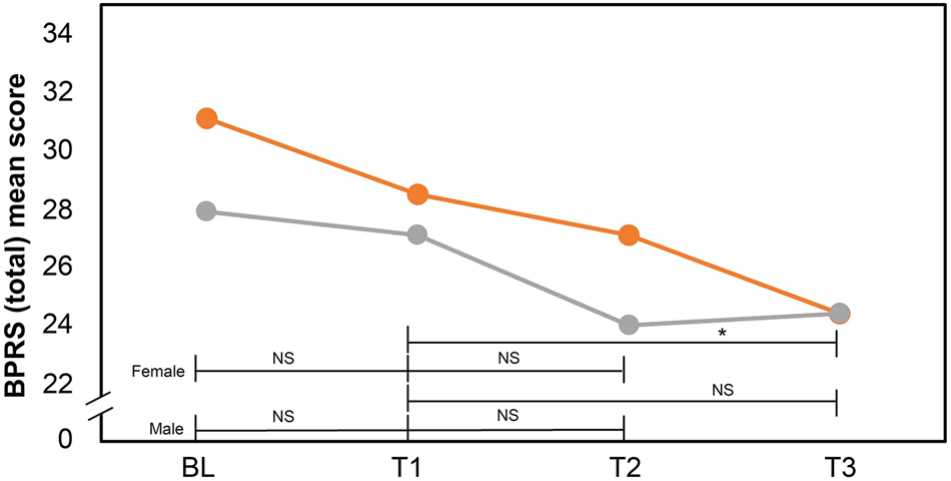
BPRS (total) mean scores of female (*n* = 10) and male (*n* = 10) participants at BL, T1, T2, and T3. After transition to circadian lighting treatment, the mean BPRS (total) scores of female participants improved significantly at T1–T3. NS, not significant; *p < .05, **p < .001. BPRS, Brief Psychiatric Rating Scale; BL, baseline (week 1); T1, test 1 (week 3); T2, test 2 (week 7); T3, test 3 (week 10).

Female participants exhibited significant psychopathological improvements under CRSL, particularly in total BPRS scores and negative symptoms from T1 to T3, as shown in Table 4 and Figure 7. In contrast, male participants did not show significant changes in BPRS scores across all assessed time points.

**TABLE 4:** MMSE, BPRS (total) and BPRS (subscale) scores of female participants (*n* = 10).

	BPRS scores of female participants (*n* = 10)						
Characteristics	BL Mean (SD)	T1 Mean (SD)	T2 Mean (SD)	T3 Mean (SD)	BL–T1 *p*	T1–T2 *p*	T1–T3 *p*
MMSE scores	29.8 (0.4)	29.1 (1.5)	29.3 (1.5)	29.3 (1.5)	.03*	.1	.2
BPRS scores (total)	31.1 (7.2)	28.5 (4. 7)	27.1 (4.7)	24.4 (5.5)	.1	.2	.005**
BPRS scores (affectivity)	10.0 (5.5)	9.8 (4.5)	8.5 (4.2)	6.9 (4.6)	.9	.2	.1
BPRS scores (positive symptoms)	5 (1.7)	4.5 (1.1)	4.9 (1.5)	4.1 (0.3)	.5	.4	.2
BPRS scores (negative symptoms)	7.5 (3.3)	6.7 (2.9)	6.7 (2.9)	4.7 (2.7)	.1	1	.002*
BPRS scores (resistance)	4.3 (1.8)	3.2 (0.4)	3.4 (0.7)	3.5 (0.8)	.1	.7	.6
BPRS scores (activation)	3.3 (0.9)	3 (0)	3 (0)	3 (0)	.3	1	1

*Note*: All data are presented as mean (SD, standard deviation), where *P*_1_ was obtained by one‐way repeated measures ANOVA; *p < .05; **p < .001.

BPRS, Brief Psychiatric Rating Scale; MMSE, Mini‐Mental State Examination; BL, baseline (week 1); T1, test 1 (week 3); T2, test 2 (week 7); T3, test 3 (week 10).

##### Cognitive function

**FIGURE 8:**
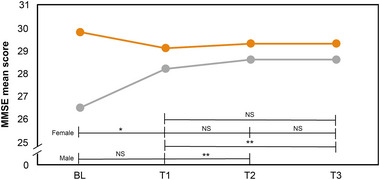
MMSE mean scores of female (*n* = 10) and male (*n* = 10) participants at BL, T1, T2, and T3. The mean MMSE scores decreased significantly at BL–T1 in female participants but improved significantly at T1–T2 and T2–T3 in male participants. NS, not significant; *p < .05, **p < .001. MMSE, Mini‐Mental State Examination; BL, baseline (week 1); T1, test 1 (week 3); T2, test 2 (week 7); T3, test 3 (week 10).

Male participants displayed a notable sensitivity to CRSL, with significant enhancements in MMSE scores from T1 to T2 and T1 to T3, indicating improved cognitive functions postintervention (Table 5 and Figure 8). Conversely, female participants did not exhibit significant cognitive changes following the CRSL intervention.

**TABLE 5:** MMSE, BPRS (total), and BPRS (subscale) scores of male participants (*n* = 10).

	BPRS scores of male participants (*n* = 10)						
Characteristics	BL Mean (SD)	T1Mean (SD)	T2 Mean (SD)	T3 Mean (SD)	BL–T1 *p*	T1–T2 *p*	T1–T3 *p*
MMSE scores	26.5 (1.8)	28.2 (2.9)	28.6 (1.8)	28.6 (1.8)	.53	.0005**	.0001**
BPR scores (total)	27.9 (5.5)	27.1 (6.7)	24 (5.1)	24.4 (5.5)	.8	.06	.09
BPRS scores (affectivity)	6.2 (3.2)	6.4 (3.7)	5.6 (2.8)	5.3 (2.7)	.8	.1	.2
BPRS scores (positive symptoms)	4.8 (1.1)	5.3 (2.4)	4.4 (1.0)	4.6 (1.3)	.4	.1	.3
BPRS scores (negative symptoms)	8.5 (3.4)	7.3 (3.7)	6.6 (3.7)	6.7 (3.0)	.2	.5	.7
BPRS scores (resistance)	3.4 (0.8)	3.7 (0.8)	3.3 (0.7)	3.7 (0.9)	.5	.1	.5
BPRS scores (activation)	3.6 (1.0)	3.4 (0.8)	3.1 (0.3)	3.1 (0.3)	1	1	.4

*Note*: All data are presented as mean (SD, standard deviations), where *P*_1_ was obtained by one‐way repeated measures ANOVA; **p < .001.

BPRS, Brief Psychiatric Rating Scale; MMSE, Mini‐Mental State Examination; BL, baseline (week 1); T1, test 1 (week 3); T2, test 2 (week 7); T3, test 3 (week 10).

These findings underscore sex‐specific responses to CRSL therapy, with females showing greater psychopathological improvements and males demonstrating enhanced cognitive responses. Throughout the study, all participants received consistent dosages of psychiatric medications, ensuring that medication influences remained constant.

## 4 DISCUSSION

This study extends the body of evidence on circadian ambient lighting's role in clinical improvement for psychiatric conditions, particularly schizophrenia. Drawing from foundational research such as Terman et al.’s (1989) work on artificial dawn and dusk's effects on seasonal affective disorder (SAD), and subsequent studies affirming artificial dawn's efficacy for SAD treatment (Avery et al., 2001; Terman & Terman, 2006), our findings highlight the potential of dynamic ambient bedroom lighting. Similar to Canazei et al. (2022), who reported positive outcomes of dawn‐dusk simulation and blue‐depleted night lighting on sleep and circadian rhythms in affective disorders, this study observed significant psychiatric symptom improvements in schizophrenia inpatients post‐CRSL intervention. Notably, the “affectivities” subscale of the BPRS showed marked enhancement, particularly with extended intervention duration, aligning with Skeldon et al.’s (2022) insights on light exposure's role in addressing sleep disturbances in schizophrenia through appropriate lighting interventions.

Furthermore, the study underscores the importance of adequate light intensity during daytime activities. Consistent with literature supporting bright light therapy for various psychiatric disorders (Aichhorn et al., 2007; Al‐Karawi & Jubair, 2016; Liu et al., 2021), our protocol involved increasing morning light intensity in patient bedrooms, complemented by outdoor activities to maximize daylight exposure. This approach aligns with Skeldon et al.’s (2022) recommendations for simple, cost‐effective strategies to enhance natural light exposure in living spaces.

### 4.1 Practical implications for light therapy settings and patient preferences

Light therapy's adaptability makes it suitable for both inpatient and outpatient settings. Inpatient settings can benefit from integrated lighting systems that simulate natural light patterns, particularly useful for patients with severe symptoms or those under constant care. Outpatient settings might utilize portable light devices that patients can use at home, aligning therapy with daily routines and personal schedules, thereby enhancing adherence and practicality.

### 4.2 Patient‐specific recommendations

Sex differences emerged as a significant response factor, with female patients showing more pronounced improvements in negative symptoms. This finding suggests that light therapy might be particularly beneficial for female patients or those exhibiting higher affective symptoms. Clinicians (Cotton et al., 2009; Seidman et al., 1997; Szymanski et al., 1995) should consider personalizing light therapy parameters, such as intensity and timing, to optimize responses based on individual patient profiles, including sex, severity of symptoms, and specific circadian disruptions.

### 4.3 Limitations and future directions

However, our study has limitations that must be acknowledged. A critical oversight was the lack of systematic assessment and reporting of potential adverse effects, such as manic symptoms, insomnia, and eye irritation, which are particularly pertinent in a psychiatric population sensitive to environmental changes. Future studies should incorporate detailed assessments of individual circadian rhythms and establish protocols for monitoring and reporting adverse effects. This could involve pre‐ and postintervention assessments specifically designed to capture changes in symptoms like insomnia, mood fluctuations, and ocular health, thereby enhancing the safety and efficacy of the intervention.

Nevertheless, the limitations of this study necessitate caution in generalizing the findings. The small sample size, the inherent challenges of conducting research in a shared inpatient setting, and the absence of a control group might undermine the robustness of the conclusions. We recognize that the Positive and Negative Syndrome Scale (PANSS) could offer a more detailed perspective on the positive and negative symptoms in schizophrenia, thereby providing additional insights into the specific domains of symptomatology. Furthermore, we acknowledge that our reliance on the Brief Psychiatric Rating Scale (BPRS) may not fully capture the complexity of symptom changes, particularly in the domain of negative symptoms. Future studies could benefit from incorporating the PANSS alongside the BPRS, contingent upon the availability of resources and the specific context of the research.

Besides, we will critically examine the use of MMSE, acknowledging its value in assessing global cognitive function but also its limitations in capturing the full spectrum of cognitive changes in schizophrenia. This will underscore the importance of a more nuanced cognitive evaluation in future studies to fully understand the impact of interventions on cognitive outcomes in this population. Additionally, the study's short duration and the inability to isolate the effects of specific lighting components (dawn and dusk simulation, blue‐depleted night lighting) call for further investigation to elucidate the long‐term impacts and precise mechanisms of CRSL interventions.

Acknowledging the feedback, our conclusion now notes the study's focus on inpatients with standardized sleep routines, limiting direct measurement of individual circadian rhythms. We recognize the value of incorporating objective measures like actigraphy in future work to deepen our understanding of circadian impacts. While adverse effects were not systematically reported, we highlight the importance of monitoring such outcomes in subsequent research, particularly focusing on cognitive and mental health improvements.

Despite these limitations, this proof‐of‐concept study contributes valuable insights into circadian lighting's therapeutic potential for schizophrenia, highlighting the need for further research into optimal settings and configurations to maximize benefits across diverse patient populations. This study highlights CRSL systems’ efficacy in improving schizophrenia inpatients' psychiatric symptoms, underscoring the potential for circadian lighting in psychiatric care. While focused on inpatients, future research should examine outpatient adaptability and refine these interventions for broader application.

## AUTHOR CONTRIBUTIONS

**Ya‐Chi Tsai**: Conceptualization; methodology; investigation; data curation; formal analysis; visualization; writing—original draft; project administration; resources. **Jwo‐Huei Jou**: Validation; writing—review and editing; supervision. **Ching‐Chi Hsu**: Data curation; methodology; validation; resources; investigation. **Ming‐Chang Shih**: Visualization; formal analysis; resources. **Luke The**: Methodology; formal analysis; visualization. **Dipanshu Sharma**: Writing—review and editing; data curation; resources.

## FUNDING

This research received no specific grant from any funding agency in the public, commercial, or not‐for‐profit sectors.

## CONFLICT OF INTEREST STATEMENT

The authors declare no competing interests.

### PEER REVIEW

The peer review history for this article is available at https://publons.com/publon/10.1002/brb3.70003.

## Data Availability

The authors confirm that the data supporting the findings of this study are available within the article and its supplementary materials.
